# Closing the diagnostic gap: Liquid biopsy potential to transform ovarian cancer outcomes in sub-Saharan Africa

**DOI:** 10.1097/MD.0000000000037154

**Published:** 2023-02-02

**Authors:** Emmanuel Kokori, Gbolahan Olatunji, Ikponmwosa Jude Ogieuhi, Ifeanyichukwu Muogbo, David Isarinade, Bonaventure Ukoaka, Irene Ajayi, Chidiogo Ezenwoba, Owolabi Samuel, Habeebat Nurudeen-Busari, David B. Olawade, Nicholas Aderinto

**Affiliations:** a Department of Medicine and Surgery, University of Ilorin, Ilorin, Kwara State, Nigeria; b Department of Medicine, Siberian State Medical University, Ilorin, Nigeria; c Department of Medicine, Ladoke Akintola University of Technology, Ogbomosho, Nigeria; d Department of Internal Medicine, Asokoro District Hospital, Abuja, Nigeria; e Department of Medicine and Surgery, Afe Babalola University, Ado-Ekiti, Ekiti; f Department of Medicine, Lagos State Health Service Commission, Lagos, Nigeria; g Department of Allied and Public Health, School of Health, Sport and Bioscience, University of East London, London, United Kingdom.

**Keywords:** diagnostic gap, healthcare infrastructure, late-stage diagnosis, liquid biopsy, ovarian cancer, sub-Saharan Africa

## Abstract

Ovarian cancer presents a significant health challenge in sub-Saharan Africa (SSA), where late-stage diagnosis contributes to high mortality rates. This diagnostic gap arises from limited resources, poor healthcare infrastructure, and a lack of awareness about the disease. However, a potential game-changer is emerging in the form of liquid biopsy (LB), a minimally invasive diagnostic method. This paper analyses the current diagnostic gap in ovarian cancer in SSA, highlighting the socio-economic, cultural, and infrastructural factors that hinder early diagnosis and treatment. It discusses the challenges and potential of LB in the context of SSA, emphasizing its cost-effectiveness and adaptability to resource-limited settings. The transformative potential of LB in SSA is promising, offering a safer, more accessible, and cost-effective approach to ovarian cancer diagnosis. This paper provides recommendations for future directions, emphasizing the need for research, infrastructure development, stakeholder engagement, and international collaboration. By recognizing the transformative potential of LB and addressing the diagnostic gap, we can pave the way for early detection, improved treatment, and better outcomes for ovarian cancer patients in SSA. This paper sheds light on a path toward better healthcare access and equity in the region.

## 1. Introduction

Ovarian cancer is a significant global health concern, and its impact is particularly pronounced in sub-Saharan Africa (SSA). In 2020, SSA witnessed almost 18,000 cases of ovarian cancer, resulting in 13,000 deaths.^[1]^ This accounted for 2.2% of all cancer cases in the region, with ovarian cancer ranking as the fourth most common neoplasm among women.^[1]^ The region faces unique challenges in the diagnosis and management of ovarian cancer, leading to a considerable diagnostic gap.

The severity of this diagnostic gap is exemplified by the high mortality rates associated with late-stage ovarian cancer diagnoses.^[2]^ Often, the disease remains undetected until it reaches advanced stages, limiting the effectiveness of treatment and resulting in poor survival rates.^[3]^ Several factors contribute to this diagnostic gap in SSA. Limited access to ovarian cancer screening and diagnostic tools is widespread in many regions, making early detection a formidable challenge.^[3]^ Additionally, the healthcare systems in SSA often lack the necessary resources and infrastructure for timely and accurate diagnosis.^[4]^ Furthermore, a general lack of awareness about the symptoms and risks associated with ovarian cancer contributes to delayed diagnosis.^[4]^

Traditional diagnostic methods commonly used in SSA, such as tissue biopsies, are invasive, costly, and require specialized medical facilities and expertise.^[5]^ These barriers further impede early detection efforts. However, there is hope in the form of liquid biopsy (LB), an emerging noninvasive diagnostic technique. LB involves analyzing specific markers or genetic alterations related to cancer in a patient blood or other bodily fluids.^[6]^ This innovative approach has the potential to revolutionize cancer detection, including ovarian cancer, by providing a less invasive, more accessible, and cost-effective diagnostic method.^[6]^ Despite its promise in various parts of the world, the application of LB in ovarian cancer diagnostics and its potential impact on SSA remains underexplored.^[7]^

There is a pressing need to examine the feasibility and implications of incorporating LB into the healthcare systems of SSA countries to address the diagnostic gap and improve outcomes for ovarian cancer patients. This paper explores LB potential to transform ovarian cancer outcomes in SSA. It provides an overview of the current status of ovarian cancer in the region, elucidates the concept of LB, and explores its advantages, challenges, and implications in its implementation as a diagnostic tool.

## 2. The diagnostic gap in SSA

The diagnostic gap in SSA regarding ovarian cancer is marked by a significant problem—late-stage diagnosis. This issue contributes substantially to the high morbidity and mortality rates associated with this disease, with a mere 29% 5-year relative survival rate, emphasizing the urgent need for enhanced early detection.^[8]^

**Figure 1.**
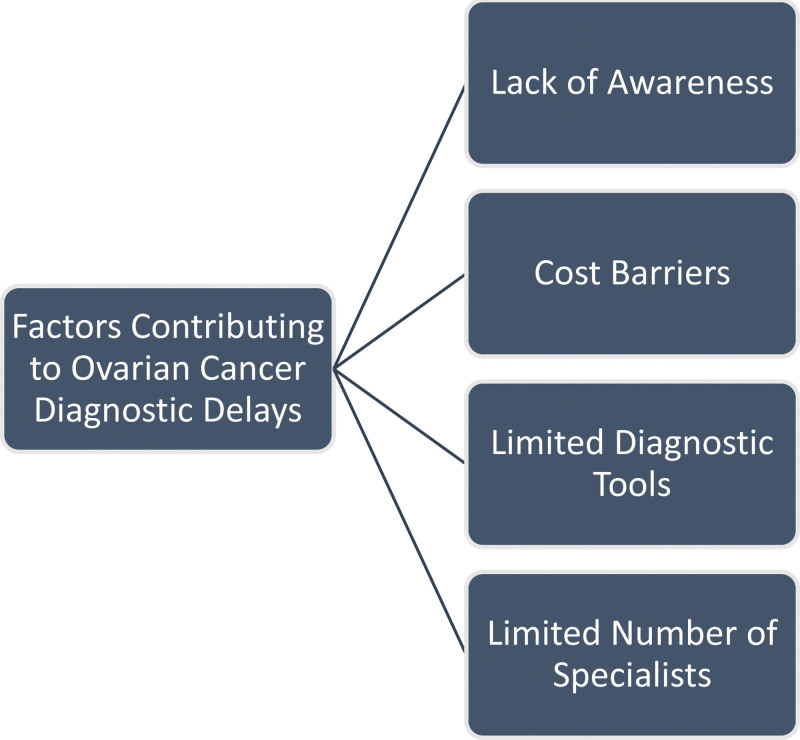
Factors contributing to ovarian cancer diagnostic delays in SSA. SSA = sub-Saharan Africa.

One key factor contributing to this diagnostic gap is the lack of awareness about ovarian cancer in the region.^[9]^ Figure 1. This lack of awareness leads to delayed symptom recognition, where nonspecific symptoms such as abdominal discomfort, bloating, or fatigue do not immediately trigger considerations of ovarian cancer among both patients and healthcare providers. Moreover, the nonspecific nature of ovarian cancer symptoms compounds the problem, as these symptoms often mimic common gastrointestinal complaints.^[9]^ Consequently, this can lead to initial misdiagnoses and referrals to unrelated specialists, ultimately resulting in diagnostic delays. Adding to this challenge, limited access to healthcare and financial constraints frequently cause individuals in SSA to postpone seeking medical attention until their symptoms reach advanced stages.^[10]^ The high healthcare costs and insufficient health insurance coverage discourage early medical consultations, permitting the disease to progress unchecked.^[10]^

The financial burden associated with cancer care is a substantial barrier in SSA. Unlike regions with more developed healthcare systems, where healthcare costs are often covered, in SSA, most healthcare expenses, including cancer-related, are paid out-of-pocket.^[11]^ This proves especially problematic in a region where a significant portion of the population lives in extreme poverty (about 33%), leading to individuals often abandoning treatments or failing to seek timely diagnosis.^[11]^ Furthermore, the strain on governments and healthcare systems in the region, as they tackle numerous public health challenges, such as malaria, HIV/AIDS, and tuberculosis, leaves limited resources and inadequate funding for cancer prevention, diagnosis, and treatment.^[12]^

Gender disparities and a lack of female education also contribute significantly to the diagnostic gap in SSA. Disparities in healthcare access and decision-making power persist between men and women in many parts of SSA, leading to delayed diagnosis, as women may not have the autonomy to seek medical attention without male consent.^[13]^ Educational inequalities in the region impact women understanding of health issues and their access to healthcare. Women with limited education may not recognize the significance of symptoms or may face cultural and social barriers to seeking medical help.^[14]^ Additionally, comorbid diseases, such as tuberculosis, often prevalent in regions where ovarian cancer is diagnosed, can mimic ovarian cancer symptoms, resulting in misdiagnoses and delayed treatments, further worsening the diagnostic gap.^[15]^

Current diagnostic methods for ovarian cancer, including monitoring of cancer antigen-125 serum levels, transvaginal ultrasound, and pelvic examination, face notable limitations. These tests have demonstrated low sensitivity in detecting ovarian cancer at an early stage, meaning that many cases remain undetected until they progress to advanced stages, reducing the effectiveness of treatment.^[16]^ Moreover, these tests often yield false-positive results, leading to unnecessary anxiety and surgical interventions, which may not be warranted, further burdening patients and healthcare systems.^[17]^ Nonetheless, LB is not 100% sensitive and specific to malignancies, and could also be found in healthy individuals and people having benign tumors.^[17]^

In addition to these challenges, the scarcity of specialists, including oncologists, pathologists, and radiologists, and the lack of essential resources like radiotherapy and chemotherapy hinder the diagnosis and treatment of ovarian cancer in SSA.^[18]^ The shortage of specialists results in prolonged waiting times and limited access to specialized healthcare services, impacting the quality and timeliness of care. The region also faces resource deficiencies, including essential medications, radiotherapy equipment, and chemotherapy drugs, further limiting the range of available treatment options and hindering comprehensive care provision. Given the invasive nature and associated risks of current diagnostic procedures, LB has emerged as a potential solution to bridge the diagnostic gap for ovarian cancer, offering a promising and noninvasive alternative to traditional diagnostic methods.

## 3. Transformative potential of LB in ovarian cancer diagnosis in SSA

LB represents a cutting-edge diagnostic tool that leverages the analysis of various components within a patient blood, including circulating tumor cells (CTCs), cell-free tumor-derived DNA, circulating microRNAs, and exosomes.^[19]^ These analytes harbor critical information concerning the presence and characteristics of cancer. LB excels in identifying genetic mutations, tumor heterogeneity, and the development of drug resistance, all of which are pivotal aspects of ovarian cancer diagnosis.^[20]^

Contrasting the traditional approach to diagnosing ovarian cancer, which often necessitates surgical biopsies or other invasive procedures for obtaining solid tissue samples, LB offers a noninvasive, low-risk alternative.^[21]^ While providing valuable insights, traditional methods come with inherent risks and limitations. Patients undergoing invasive procedures face the threat of infections, and often, the samples collected may not adequately represent the full spectrum of diseased or altered cells within the tumor.^[22]^ In contrast, LB relies on blood samples, which can be obtained with relative ease and minimal patient discomfort. These samples contain vital biomarkers, such as circulating tumor DNA, carrying genetic information specific to the tumor.^[23]^ The ability of LB to capture genetic diversity and tumor heterogeneity represents a significant advantage over traditional methods. Furthermore, developing next-generation sequencing techniques has greatly improved the sensitivity and specificity of LB, enabling the detection of even trace amounts of circulating tumor DNA.^[24]^ This advancement is pivotal in ensuring the accurate diagnosis and monitoring of ovarian cancer.

One of LB most promising and pivotal aspects, particularly in SSA, is its remarkable adaptability to resource-limited settings, addressing a critical need in a region where traditional diagnostic methods often fall short. The diagnostic gap in SSA, marked by late-stage ovarian cancer diagnoses, is significantly exacerbated by the lack of access to specialized healthcare facilities and a scarcity of highly trained personnel, both of which are typically essential for conventional diagnostic procedures.^[25]^

In regions facing these challenges, where advanced healthcare infrastructure is a rarity, LB emerges as hope for countless patients in SSA.^[26]^ Unlike conventional diagnostic methods that often mandate complex, specialized equipment, LB operates with significantly fewer demands.^[27]^ This makes it a feasible option even in healthcare settings with limited resources. The need for highly advanced machinery, often expensive and hard to come by, is notably reduced with LB. Such minimal equipment requirements ensure that even healthcare facilities with limited financial means can effectively employ this diagnostic technique, expanding its reach and impact. Similarly, LB is not dependent on cutting-edge laboratory facilities or high-tech infrastructure.^[26]^ Standard laboratory facilities, which are more prevalent and accessible in resource-limited settings, suffice to process blood samples. This compatibility with existing infrastructure means that LB can be readily incorporated into the healthcare systems of SSA without the need for costly overhauls or extensive renovations.

While traditional diagnostic methods often require a team of highly specialized healthcare professionals, LB is less dependent on such expertise.^[28]^ Given the scarcity of specialized medical personnel in many underserved regions, this is a crucial advantage. LB procedures can be conducted and interpreted by a broader range of healthcare practitioners, reducing the burden on the limited pool of specialized experts and increasing the availability of this diagnostic approach.^[29]^ Furthermore, LB seamlessly fits into various healthcare settings, ranging from well-equipped urban hospitals to mobile clinics that serve remote and rural communities.^[30]^ The adaptability of LB to various healthcare settings is invaluable in regions where access to specialized healthcare facilities is often constrained. Patients in these areas may need to undertake arduous journeys to access medical care, which can lead to delays in diagnosis and treatment. LB bridges this geographical gap, offering a means for earlier diagnosis and improved treatment outcomes.

## 4. Challenges to implementing LB in SSA

In SSA, the implementation of LB as a method for diagnosing cancer, particularly ovarian cancer, is beset with numerous challenges. Despite significant regional social, economic, and technological advances, these developments fall short compared to the Western world. Several factors contribute to the difficulties in adopting LB as a diagnostic tool for cancer in SSA.

The region needs better government policies, inadequate health infrastructure, and a notable lack of skilled healthcare personnel.^[25]^ These systemic problems hinder the smooth integration of advanced diagnostic technologies like LB into healthcare. Similarly, cost and affordability represent a primary challenge in resource-limited countries, as the estimated cost per LB test in Kenya, for example, amounts to a substantial 70000 Kenyan Shillings, roughly equivalent to $7000.^[31]^ The financial burden of such tests, which often require expensive equipment and reagents, poses a formidable barrier to widespread adoption.

Transportation and sample storage is another challenge. LB relies on isolating CTCs from peripheral blood samples, a technically challenging process, given the low concentration of CTCs in blood.^[7]^ Regions with limited access to reliable transportation and consistent electricity face significant hurdles in safely transporting and storing collected samples. Proper sample handling and storage are paramount to the success and accuracy of LB, and inadequate infrastructure can compromise the quality of results.

Patient awareness and acceptance are critical challenges in implementing LBs in resource-limited countries. Social stigma and cultural beliefs can greatly affect the perception of cancer and the acceptance of new diagnostic methods. Raising awareness and educating the public about the benefits and accuracy of LB are vital steps in overcoming these barriers. Furthermore, patient data management and privacy are crucial considerations. LB requires the management of patient data, including the storage and transmission of sensitive medical information. Ensuring data security and privacy is paramount, but healthcare systems in the region may need more infrastructure and protocols to protect patient data, posing ethical and practical challenges adequately.

## 5. Future directions and recommendations

**Figure 2.**
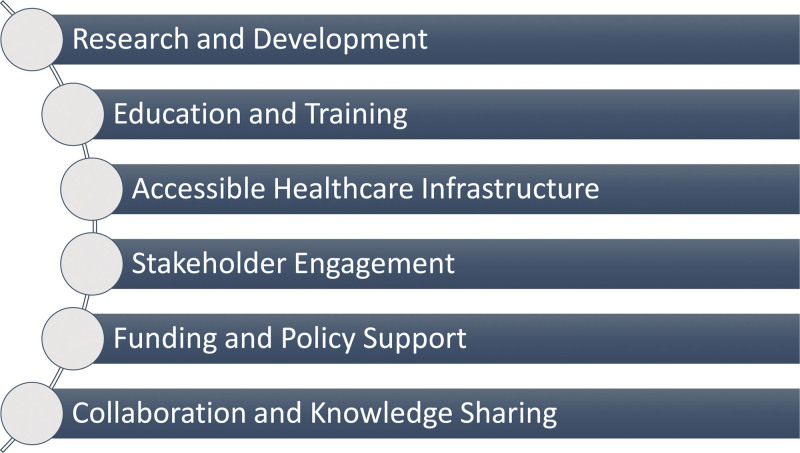
Key recommendations for liquid biopsy integration.

The journey to bridge the diagnostic gap for ovarian cancer in SSA and unleash the transformative potential of LB is multifaceted and demands a comprehensive approach. To navigate this path, several critical future directions and recommendations emerge, forming the bedrock for enhancing ovarian cancer outcomes and ushering in a new era of diagnostics in SSA—Figure 2.

*Research and development:* The prospect of LB application in SSA for ovarian cancer diagnosis is promising. However, it is imperative to acknowledge that there a unique set of challenges and variables within the region. Hence, it is recommended that concerted efforts in research and development be undertaken. Collaborations between local researchers, healthcare institutions, and international partners should be fostered to conduct rigorous clinical trials that validate LB efficacy in the SSA context. These trials must account for the genetic diversity and environmental factors specific to the region. They can serve as a solid foundation for the customization of LB for SSA, ensuring its reliability and effectiveness.

*Education and training:* Implementing LB into the healthcare systems of SSA necessitates a robust educational framework. Establishing comprehensive educational and training programs to equip pathologists, clinicians, and healthcare providers with the requisite skills to interpret and apply LB results competently is vital. Academic institutions and professional organizations should collaborate to create specialized training modules tailored to SSA unique needs. Certification programs that reflect local conditions are essential to ensure the successful integration of LB.

*Accessible healthcare infrastructure:* Overcoming infrastructure challenges is central to adopting LB in SSA. Access to reliable electricity, advanced laboratories, and essential equipment is pivotal for implementing LB, especially in regions with frequent power outages and resource constraints. Collaboration between governments, international organizations, and private sectors addresses these infrastructure gaps. Efforts should be focused on building or upgrading healthcare infrastructure, eliminating this fundamental barrier to LB success.

*Stakeholder engagement:* Effective stakeholder engagement is the linchpin of LB adoption in SSA. It necessitates collaboration with patient advocacy groups, local communities, and healthcare providers to foster awareness and acceptance of LB. This engagement must be informed by a deep understanding of the cultural beliefs, social stigma, and privacy concerns surrounding cancer diagnosis in SSA. A bottom-up approach, involving patients and communities in the decision-making process, is essential. By addressing the unique sociocultural factors, LB can achieve wider acceptance and seamless integration into SSA diverse healthcare landscape.

*Funding and policy support:* Advocacy for funding and policy support represents a critical aspect of advancing LB in SSA. Robust advocacy efforts should focus on securing financial support from government bodies, international agencies, and philanthropic foundations. Policymakers need to be sensitized to the significance of LB in national cancer control policies. To ensure the sustained development and integration of LB, it is essential to collaborate with international partners and organizations to secure grants and funding specifically designated for LB research and implementation.

*Collaboration and knowledge sharing:* Collaboration is the adhesive that binds these recommendations into a cohesive action plan. Within SSA, countries should actively share experiences, best practices, and research findings related to LB implementation. These exchanges can catalyze progress and circumvent common pitfalls. Moreover, partnering with international organizations like the World Health Organization can provide valuable support and expertise. Seeking collaboration with the European Commission for financial assistance can prove pivotal in obtaining funds for integrating LB technology within the healthcare systems of SSA.

In embracing these future directions and recommendations, the ambition of revolutionizing ovarian cancer diagnosis and treatment in SSA through LB can be effectively actualized. Together, these steps pave the way for closing the diagnostic gap, facilitating early detection, and ultimately elevating the treatment and survival rates of ovarian cancer patients in the region. The success of this transformative journey hinges on a multifaceted, coordinated effort that spans research, education, infrastructure, advocacy, and international collaboration. By embracing these core principles, the future of ovarian cancer diagnosis in SSA can be forever altered.

## 6. Conclusion

In SSA, the diagnostic gap in ovarian cancer looms large, casting a shadow on the lives of countless women. Late-stage diagnoses, scarce resources, and social complexities have compounded the burden of this disease. However, the transformative potential of LB can reshape the landscape of ovarian cancer diagnosis in this region. Our exploration into the diagnostic gap and LB potential to bridge it illuminated a path toward a brighter future for ovarian cancer patients in SSA. The key takeaways from this paper can guide stakeholders, researchers, policymakers, and healthcare providers toward a comprehensive strategy for change.

The adaptation of LB in SSA is not without its challenges. From research and development customized to the region unique needs to accessible healthcare infrastructure and comprehensive educational and training programs, our recommendations outline a roadmap for success. Stakeholder engagement, societal awareness, and policy support are the cornerstones of realizing LB potential in SSA. This transformative journey hinges on a multifaceted, coordinated effort that spans research, education, infrastructure, advocacy, and international collaboration. By embracing these core principles, the future of ovarian cancer diagnosis in SSA can be forever altered. In closing the diagnostic gap, the lives of countless women will be impacted positively. LB offers the potential to revolutionize early detection, enhance treatment outcomes, and ultimately save lives. It represents a beacon of hope, shining light in SSA darkest corners of the ovarian cancer landscape.

## Author contributions

**Conceptualization:** Emmanuel Kokori, Gbolahan Olatunji, Nicholas Aderinto.

**Writing – original draft:** Emmanuel Kokori, Gbolahan Olatunji, Ikponmwosa Jude Ogieuhi, Ifeanyichukwu Muogbo, David Isarinade, Bonaventure Ukoaka, Irene Ajayi, Chidiogo Ezenwoba, Owolabi Samuel, Habeebat Nurudeen-Busari, David B. Olawade, Nicholas Aderinto.

**Writing – review & editing:** Emmanuel Kokori, Gbolahan Olatunji, Ikponmwosa Jude Ogieuhi, Ifeanyichukwu Muogbo, David Isarinade, Bonaventure Ukoaka, Irene Ajayi, Chidiogo Ezenwoba, Owolabi Samuel, Habeebat Nurudeen-Busari, David B. Olawade, Nicholas Aderinto.
